# Modified perineal linear stapler resection for external rectal prolapse

**DOI:** 10.1016/j.amsu.2020.03.011

**Published:** 2020-04-13

**Authors:** Osama H. Khalil, Tamer A.A.M. Habeeb, Bassem M. Sieda

**Affiliations:** Department of Surgery, Faculty of Medicine, Zagazig University, Egypt

**Keywords:** Constipation, Fecal incontinence, Rectal prolapse, Perineal linear stapler resection

## Abstract

**Background:**

rectal prolapse can cause bleeding and fecal incontinence that affects the life quality of patients. The treatment of external rectal prolapse is surgical. There are many procedures (abdominal or perineal) that can be used depending on the severity of the condition and patient tolerability for operation. In this study, a simple safe procedure is used for the treatment of the rectal prolapse in old, fragile and comorbid patients who cannot withstand the major surgeries and the risk of long-duration anesthesia.

**Methods:**

from December 2016 to July 2019, 36 elderly comorbid patients with rectal prolapse were involved in this study which is performed in the GIT surgery unit of Zagazig University Hospital. A modified linear stapler resection technique is used for the rectal prolapse. Postoperative follow up was done for one year to evaluate the functional outcome, operative time, hospital stay duration and complications.

**Result:**

this study was conducted on 36 patients; The median age was 75 years (range 48–95). The postoperative complication rate was 11.1%. The median operative time was 25 min and 4 days for the hospital stay. Fecal incontinence improved in more than 90% of patients and constipation disappeared in 66% of total constipating patients.

**Conclusion:**

The modified perineal linear stapler resection for external rectal prolapse is a good, easy, rapid treatment for elderly comorbid patients with good functional outcomes.

## Highlights

• Treatment of complete external rectal prolapse in elderly comorbid patients is a challenge.
• This operation decreased the coast by using 1 stapler and 2 cartridges.
• The main obstacles for this operation is the high cost of stapler used in it.
• The recent modification reduced the coast by using one linear stapler and 2 cartridges.

## 1 Introduction

The perineal stapled prolapse resection (PSP) was used for the first time in 2008. It showed good promising results regarding functional outcome and complications [1]. In old patients with comorbidity, the perineal approach like Delorme's operation and the Altemeire operation are the most common procedures for rectal prolapse as these patients are not suitable for the abdominal approach. The shorter operating time of (PSP) is the main advantage over other techniques [2]. The other advantages of (PSP) are large median circumference with less postoperative capacity reduction and less anastomotic stenosis in comparison to circular stapler used in the modified perineal rectosigmiodectomy [3]. The higher cost of (PSP) is the main obstacle for usage so in this study (PSP) was done at a lower cost by using a simple modification (only one reloadable linear stapler with 2 cartridges).

## 2 Methods

The study was done in the GIT Unit of the General Surgery Department of Zagazig University Hospital in the period from December 2016 to July 2019. The hospital Institutional Review Board approved the study protocol. Thirty-six old, comorbid or short life expectancy patients were included in the study sample. They were evaluated for PSP. Informed consent was signed by all patients or first-degree relatives after full discussion of the advantage and disadvantages of the operation. Preoperative bimanual examination was done for rectal prolapse to rule out enterocele or cystocele and this was confirmed by MRI. The routine preoperative evaluation was done for all patients (physical examination, complete blood tests, ECG and chest x-ray). Bowel preparation was done for all patients.

Regarding bowel function, all patients were evaluated for fecal incontinence by Wexner score [4] and for constipation by Rome II criteria [5]. Prophylactic intravenous cephalosporin and metronidazole was given 1 h before the operation. The operation was done under spinal anesthesia in the lithotomy position with slight Trendelenburg to prevent trapping of abdominal organs between walls of the rectum. All operations were done by the same surgical members of the unit. Hospital stay, Intraoperative and postoperative complications were recorded. All patients started oral fluids on the second day. Follow up was done for patients every 1,3,6,9 and 12 months in the outpatient clinic.

### 2.1 Surgical technique

**Fig. 1:**
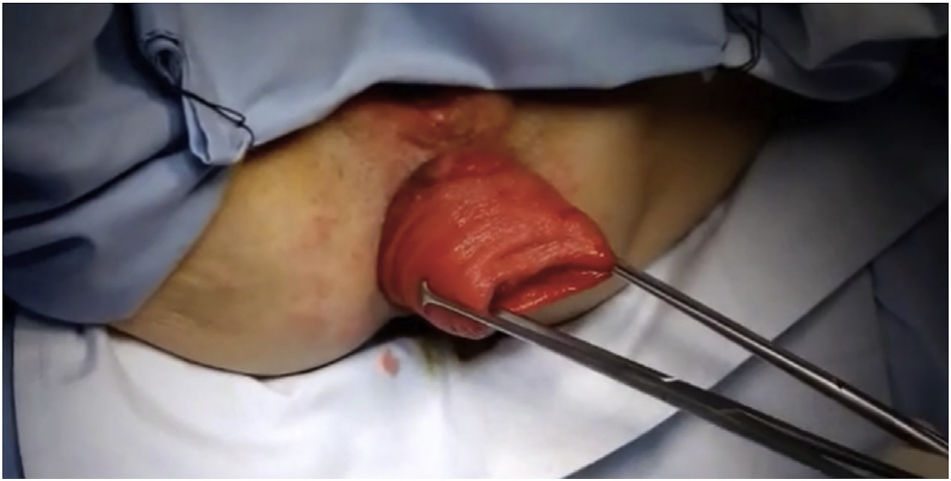
Rectal prolapse.

**Fig. 2:**
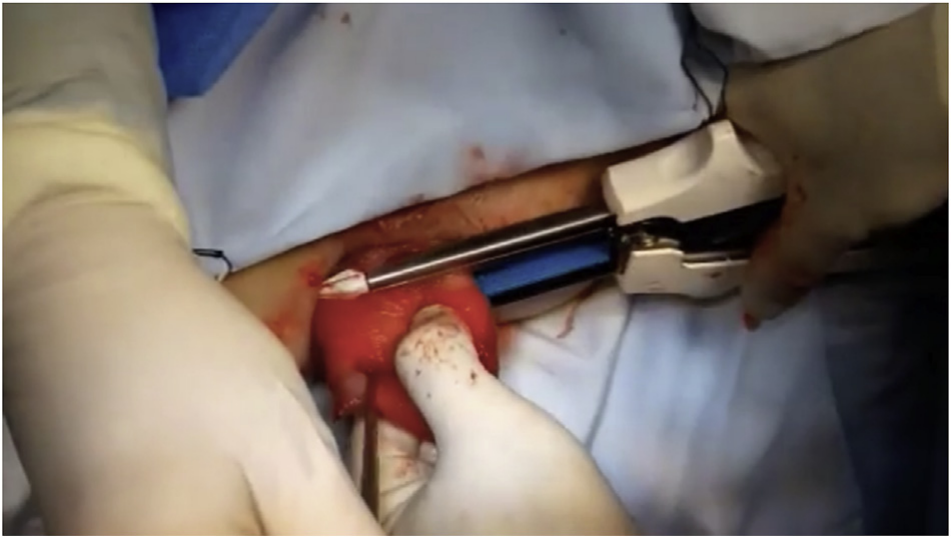
Linear stapler passing through tunnel to cut anterior aspect of prolapse.

**Fig. 3:**
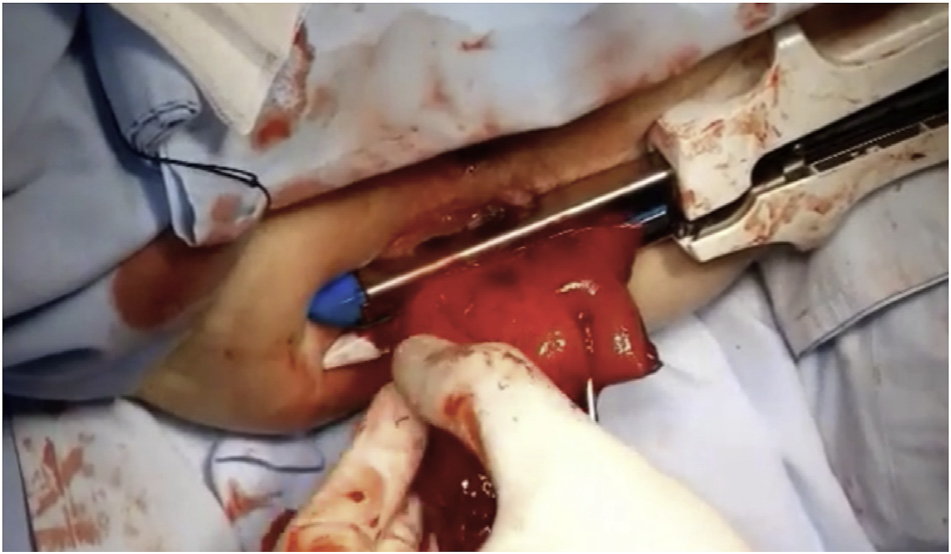
Linear stapler cut posterior aspect.

**Fig. 4:**
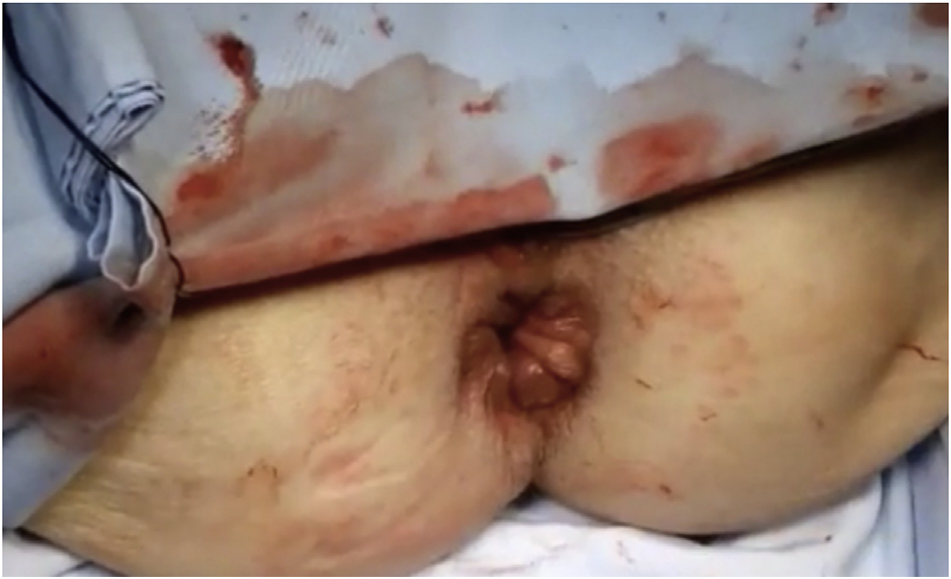
The prolapsed rectum falls back the prolapse.

After anesthesia and patient position, the prolapse was pulled out (Fig. 1) Two vertical incisions were made in both the inner and outer walls of the prolapse (at 3and 9 o'clock)1–2 cm in size approximately 2 cm above the dentate line. A reloadable linear cutter stapler (GIA 100 mm Covidien, Mansfield, Mass., USA) is used to pass through the tunnel to cut the anterior aspect of the prolapse (Fig. 2**)**. Then, after reloading the stapler by the new cartridge, it was used to do the same for the posterior wall and the prolapsed rectum was completely transected (Fig. 3). The surgeon ensures that the stapler did not fire at the dentate line to avoid post-operative pain. Multiple 3-0 PDS intermittent full-thickness sutures were done for hemostasis. The prolapsed rectum falls back into place spontaneously(Fig. 4).

### 2.2 Statistical analysis

The statistical analysis was performed by the Statistical Package for the Social Sciences (SPSS) program, version 21. The data presented in tabular form showing the frequency and relative frequency distribution of different variables of the study. P values of 0.05 were used as a cut off point for the significance of the *Chi-square* statistical test.

## 3 Results (Table 1)

**Fig. 5:**
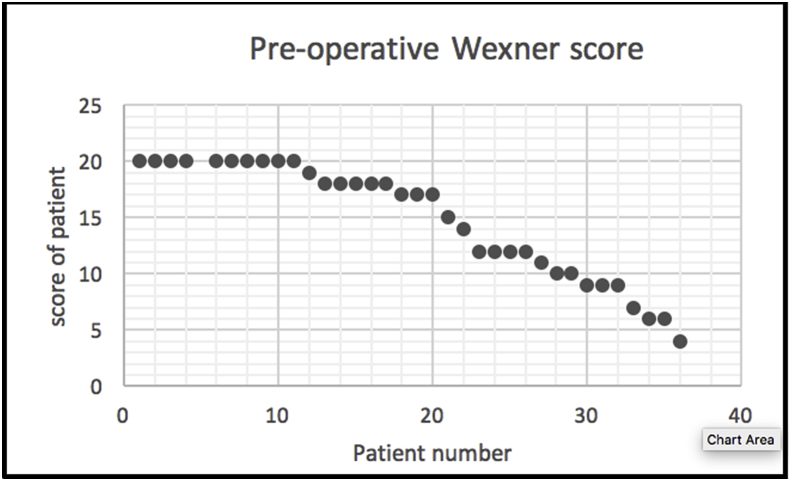
Pre-operative Wexner score.

**Fig. 6:**
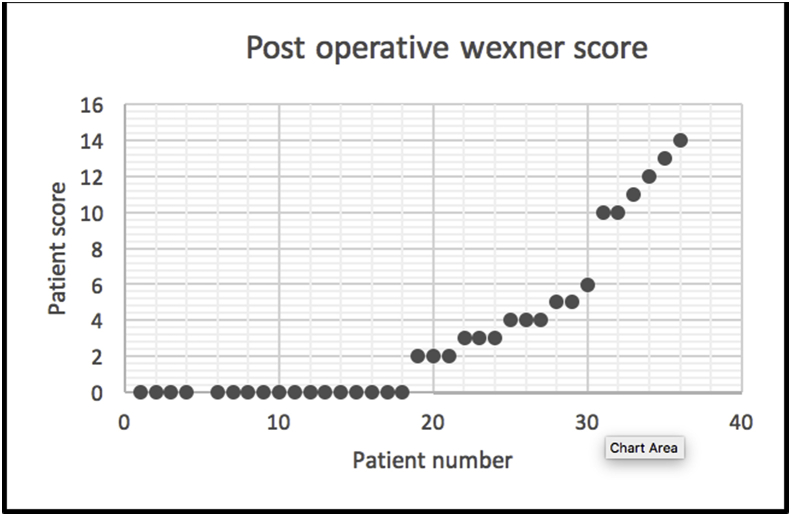
Post operative wexner score.

**Fig. 7:**
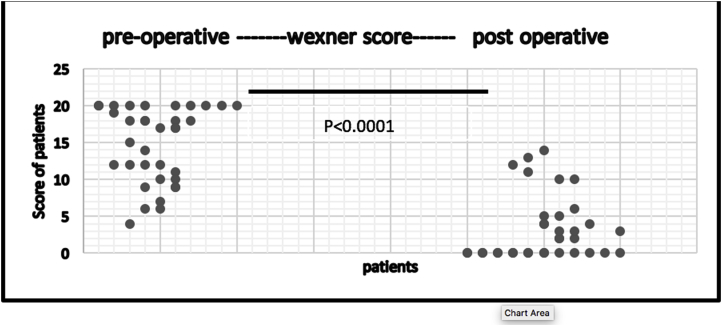
The significant difference between post and pre-operative Wexner score.

The modified PSP was performed in 36 patients. The median age was 75 years (range 48–95). The peak of incidence was between (66 and 80 years) (p-value <0.0001). Six of the patient were males (16.6%) and 30 females (83.4%) (p-value <0.00001). Five patients (13.8%) were suffering from recurrent rectal prolapse after previous different operative procedures. Thirty-one patients (86.1%) represented in the GIT unit for the first time with external rectal prolapse (p value < 0000.1). According to the American Society of Anesthesiologists(ASA), the perioperative grade for the patients was from grade I to III with 22 patients (61.1%) of grade II (p-value<0.00001). Spinal anesthesia succeeded in 26 patients (72.2%), the other 10 patients (27.3%) underwent general anesthesia. The modified PSP was done and completed in all cases without any intraoperative complications. The median of operative time was 25 min (range 15–55) and 25 cases (69.4%) were completed within (15–30min) (p-value<0.00001). 2 cartridges were only used in all cases with interrupted (3-0 PDS) sutures. The resected mass weight varies from 25 to 180 g with median 55 g. Four patients (11.1%) showed early postoperative complications within 30 days after surgery. Two cases (5.5%) were suffering from anal bleeding and were controlled by anal pack and conservative measures. One case (2.7%)showed upper respiratory infection and controlled by an antibiotic. One case (2.7%) was suffering from urinary tract infection and was controlled also by an antibiotic. The median of hospital stay was 4 days (range from 2 to 12 days) and most of the patients were discharged before one week (p-value<0.0001). The perioperative median Wexner score of fecal incontinence was 17 with rang (4-20), after the operation it became 1 and range was (0-14). Fecal incontinence disappeared in 33 patients (91.6%) Fig. 5, Fig. 6, Fig. 7. Nine patients (25%) were suffering from preoperative constipation which improved in 6 patients (66%out of 9) after surgery.

**Table 1:** Distribution of patient demographics.

Variable		Frequency	Percentage	P-value
Gender	Male	6	16.7%	P < 0.0001*
	Female	30	83.3%	
Age	48–65	3	8.3%	P < 0.0001*
	66–80	23	63.9%	
	>80	10	27.8%	
Presentation of patient	Recurrent	5	13.9	P < 0.00001*
	Non- recurrent	31	86.1	
Diseases duration	1–2 m	25	69.4%	P < 0.00001*
	3–5 m	6	16.7%	
	>5	5	13.9%	
Constipation	Constipating	9	25%	P < 0.0001*
	Non - constipating	27	75	
ASA score	Grade I	10	27.8%	P < 0.00001*
	Grade II	22	61.1%	
	Grade III	4	11.1%	
Time to complete	15–30	25	69.4%	P < 0.00001*
	30–45	6	16.7%	
	45–55	5	13.9%	
Mass size	25–75	9	25%	P < 0.00001*
	75–125	24	66.7%	
	125–180	3	8.3%	
Hospital stay	2–6 days	23	63.9%	P < 0.00001*
	6–10days	10	27.8%	
	10–12days	3	8.3%	

*Chi-square test was used* P-value <0.05 is significant*.

## 4 Discussion

The abdominal rectopexy is the treatment of choice of complete rectal prolapse with a low recurrence rate and good continence in most of the patients (50–88%). In the elderly comorbid patients, the abdominal approach is not suitable for them, the perineal approach like perineal rectosigmiodectomy and the Delorme operation are a good choice with fewer complications and high recurrence rates [6].

In 2007, a novel surgical technique (the perianal stapled prolapse resection) (PSPR) was introduced and completed in 12 patients without major complications. The advantage of this operation is easy, fast, short hospital stay, early recovery [1,2]. Although the high cost of the staplers used in this technique, it is still nearly equal to the cost of laparoscopic rectopexy including mesh, tackers, operative charges and anesthesia charges [7,8].

In 2011, a modification of the PSPR was done in a case report by using only one linear stapler with 2 cartridges for resection of rectal prolapse in old patients without any complications or short-term recurrence [9].

In this studied group, 30 females repented 83.3% of total patients with a significant difference between males and females. In the adult population, the male-to-female ratio is 1:6 regarding the incidence of rectal prolapse. Although in the adult population, women account for 80–90% of cases [10]. In another study of Hetzer and his colleges which was done on 32 persons for PSP evaluation, females represented 93% of the total studied group of patients [2].

Evaluation of the new modification of PSP depends on the functional results of the surgery including incontinence, constipation, and pudendal nerve injury. In this study, the median Wexner score of fecal incontinence was 17 before the operation and became 1 after the operation with a significant difference as seen in Fig. 5, Fig. 6, Fig. 7. Many studies record different grades of fecal incontinence from 30 to 100% [11]. This study is matching the study of Hetzer et al.; as his study improvement of incontinence was more than 90% of patients [2]. The other surgical procedures like laparoscopic rectopexy, one study reported improvement of fecal incontinence in 71% of the studied group [12]. Heath et al. used laparoscopic suture rectopexy without resection with no cases of recurrence in their 25 patients' case series, and none had worsening incontinence following the operation [13].

Constipation was successfully treated in this study with a success rate of 66% of constipating patients. This outcome is matching the other studies of Hetzer et al. and Bajaj et al. Constipation is a common complication of transabdominal rectopexy up to 50% [14]. Also, it may worsen after abdominal procedures [15,16]. Resection was favored by other studies to avoid post-operative constipation at least up to 50% but the mortality rate increased to 10–15% due to postoperative complications [[17], [18], [19], [20], [21]].

In this technique, nerve damage is impossible as no dorsal mobilization of the rectum as in transabdominal rectopexy. The patients showed good control of micturition and sexual function which is matching the studies done on the same technique and its modification [1,7,9]. Nerve injury can happen in 17% of patients underwent transabdominal rectopexy [221]. This percentage decreased after the modification of the technique done by D'Hoor et al. to 7% and little recurrence rate 3.7% [23]. Eighty percent of the patients were satisfied postoperatively and this matching the other studies in which (77–91%) of patients assed the PSP operation as a good choice with good outcome [2,7].

## 5 Conclusion

Treatment of complete external rectal prolapse in elderly comorbid patients is a challenge. The PSP technique is a good choice due to short operative time, less blood loss, no abdominal exploration, easy technique, short learning curve, rapid recovery, and short hospital stay. The main obstacle for this operation is the high cost of stapler used in it. The recent modification reduced the coast by using one linear stapler and 2 cartridges. Long term follows up may be needed for more evaluation of recurrence rate and functional outcome.

## Ethical approval

Ethical approval was given by Zagazig university – faculty of medicine institutional review board with number IR-170133-1.

## Sources of funding

No sources of funding.

## Author contribution

Osama Khalil: study design – operative procedure – data collection.

Tamer Habeeb: data collection – operative procedure – data analysis.

Basem Sediah: operative procedure – data analysis – writing.

## Research registration number

1. Name of the registry: clinical trial gov
2. Unique Identifying number or registration ID: NCT04184310
3. Hyperlink to the registration (must be publicly accessible): https://register.clinicaltrials.gov/prs/app/template/AdminEmail.vm?uid=U0003ROM&ts=6&cx=-6ayetk

## Guarantor

Osama Khalil.

## Provenance and peer review

Not commissioned, externally peer reviewed.

## Declaration of competing interest

No conflicts of interest among authors.
